# Artificial intelligence-driven assessment of sarcopenia in orthopedic geriatrics: technical progress and clinical implications

**DOI:** 10.3389/fendo.2026.1779448

**Published:** 2026-03-18

**Authors:** Tengbo Pei, Yutian Lei, Yufang Gao, Minjie Zhang, Tao Xu, Weina Yang, Qifu Wen, Qiang Liu

**Affiliations:** 1 Department of Medical Laboratory, Xianyang Central Hospital, Xianyang, Shaanxi, China; 2 Department of Orthopedics I, Xi’an Daxing Hospital, Xi’an, Shaanxi, China; 3 Department of Human Anatomy, Histology and Embryology, School of Basic Medical Sciences, Xi’an Jiaotong University Health Science Center, Xi’an, Shaanxi, China; 4 Department of Orthopedics, Xianyang Central Hospital, Xianyang, Shaanxi, China

**Keywords:** artificial intelligence, clinical decision support systems, deep learning, geriatric orthopedics, opportunistic screening, osteosarcopenia, sarcopenia

## Abstract

Sarcopenia, a progressive skeletal muscle disorder characterized by the loss of muscle mass and function, represents a significant challenge in geriatric orthopedics, with prevalence reaching as high as 48.7% in surgical populations. It is strongly associated with increased risks of falls, secondary fractures, postoperative complications, and mortality. Despite its clinical importance, traditional diagnostic methods like Dual-energy X-ray Absorptiometry (DXA) and Bioelectrical Impedance Analysis (BIA) are often impractical in acute orthopedic settings due to patient immobilization, positioning constraints, and postoperative fluid imbalances. This narrative review aims to summarize how the emergence of artificial intelligence (AI), particularly deep learning, addresses these gaps by enabling automated, high-throughput opportunistic screening from routine clinical imaging. Convolutional neural networks achieve expert-level segmentation of muscle quantity and quality, with Dice similarity coefficients often exceeding 0.94. AI-derived metrics serve as robust independent predictors for adverse surgical outcomes, including prolonged length of stay and infection, as well as functional recovery and one-year mortality. By integrating these metrics into Clinical Decision Support Systems (CDSS) and Electronic Medical Records (EMR), AI facilitates a paradigm shift from reactive fracture management to proactive prevention through automated “zero-click” alerts and multidisciplinary intervention pathways. While significant challenges regarding technical standardization, biological variability, and model interpretability persist, AI-driven assessment is transforming geriatric orthopedic care from subjective evaluation toward precise, objective quantification.

## 1 Introduction

### 1.1 Sarcopenia in the aging population: definition and its devastating impact on geriatric orthopedic outcomes (falls, frailty, and mobility loss)

Sarcopenia is a progressive skeletal muscle disorder characterized by loss of muscle mass, strength, and function (1). The 2019 European Working Group on Sarcopenia in Older People 2 (EWGSOP2) consensus defines sarcopenia by reduced muscle strength plus decreased muscle mass or quality, while physical performance indicates severity (2). The Sarcopenia Definition and Outcomes Consortium further recommends including both low grip strength and slow gait speed (1). Sarcopenia affects around 10–16% of older adults worldwide, with higher rates in clinical settings (3). In geriatric orthopedics, prevalence reaches 37% in hip fracture patients and up to 48.7% in total knee arthroplasty candidates (4).

Sarcopenia markedly worsens outcomes in elderly orthopedic patients. It increases fall risk 1.60-fold and fracture risk 1.84-fold (5). After surgery, patients experience poorer recovery and higher mortality, particularly in emergency procedures (6). Sarcopenia is also linked to greater complications and inferior knee function following total knee arthroplasty (4). As a key component of frailty, it doubles frailty risk (7). Motor impairment is central to disability and mortality associations rather than muscle mass loss alone (8).

### 1.2 The “osteosarcopenia” connection: the synergistic relationship between bone and muscle health in orthopedic patients

**Figure 1:**
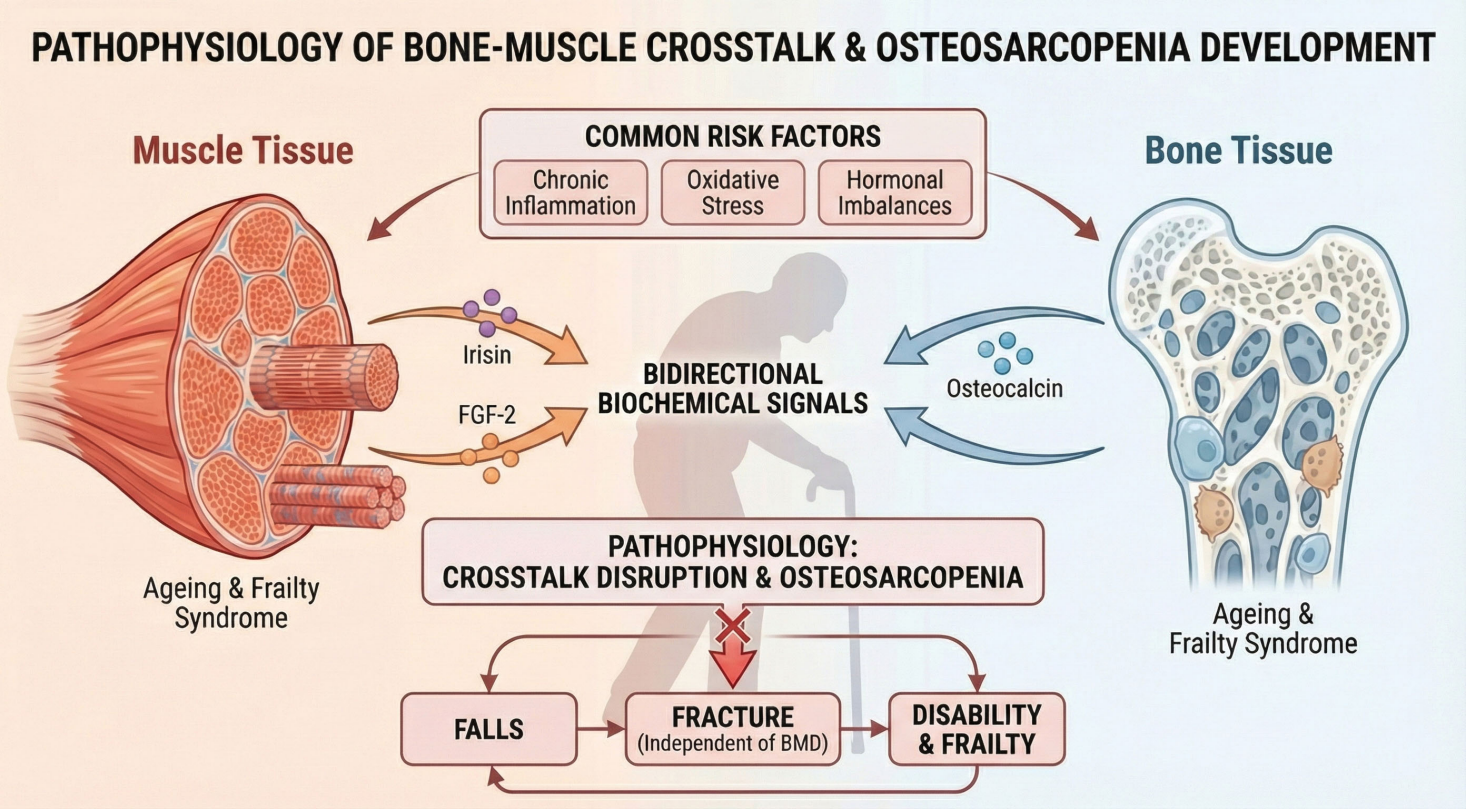
Pathophysiological mechanisms of bone-muscle “crosstalk” and the development of osteosarcopenia. This schematic illustrates the bidirectional biochemical signaling and shared systemic risk factors governing musculoskeletal homeostasis. Muscle tissue acts as an endocrine organ, releasing myokines such as irisin and fibroblast growth factor-2 (FGF-2) to promote bone formation, while bone-derived osteocalcin supports muscle anabolism. The disruption of this crosstalk, driven by chronic inflammation and oxidative stress, leads to a vicious “falls-fracture-disability” cycle.

Osteoporosis is a systemic skeletal disease characterized by low bone mass and microarchitectural deterioration of bone tissue, leading to increased bone fragility. Osteosarcopenia denotes the coexistence of osteoporosis and sarcopenia, a combined syndrome in which parallel bone and muscle loss jointly magnify clinical risk (1). Both tissues show coordinated decline with aging, driven by chronic inflammation, oxidative stress, and hormonal disturbance (1). Their interaction extends beyond mechanical loading to bidirectional biochemical signaling through myokines and osteokines (9). Muscle-derived mediators such as irisin and fibroblast growth factor-2 regulate bone remodeling, while bone-derived osteocalcin supports muscle anabolism (10). Disruption of this muscle–bone crosstalk is now recognized as central to osteosarcopenia pathogenesis (10). In orthopedic populations, osteosarcopenia confers higher risks of falls, fractures, and mortality than either condition alone (11). It is particularly relevant in surgical patients, predicting poorer recovery, more complications, and higher readmission after procedures including total hip and knee arthroplasty (12). Notably, sarcopenia increases fracture risk independent of bone mineral density, with muscle strength and physical performance emerging as key predictors of fragility fractures (13). The intricate biochemical crosstalk between bone and muscle and the resulting clinical cascade—from signaling disruption to increased fracture vulnerability—are visually summarized in **Figure 1**.

### 1.3 Clinical diagnostic gaps: why traditional methods (bioelectrical impedance analysis, DXA) are often impractical in acute orthopedic settings (e.g., hip fractures)

Dual-energy X-ray absorptiometry (DXA), a widely accepted reference method for quantifying body composition—including appendicular lean mass—based on the differential attenuation of low-dose X-rays, is difficult to implement in acute orthopedic care (14). Transporting critically ill or immobilized trauma patients to DXA facilities is rarely prioritized, and mobility restrictions after hip fracture prevent accurate positioning (14). DXA also requires dedicated equipment, trained staff, and extra scanning time, limiting use in emergency settings (14). Acute pain, surgical hardware, and positioning constraints further reduce accuracy (15). Bioelectrical impedance analysis (BIA), which estimates body composition by measuring the resistance of body tissues to a weak electrical current, although portable, is highly sensitive to fluid imbalance, fever, medications, and hydration changes common in postoperative elderly patients (16). Accurate assessment requires standardized conditions rarely achievable in acute care, particularly in the presence of implants (17). As a result, sarcopenia remains frequently underdiagnosed in hip fracture patients despite prevalence exceeding 30–40% (18).

### 1.4 Machine learning for initial screening: simplifying muscle mass estimation

**Figure 2:**
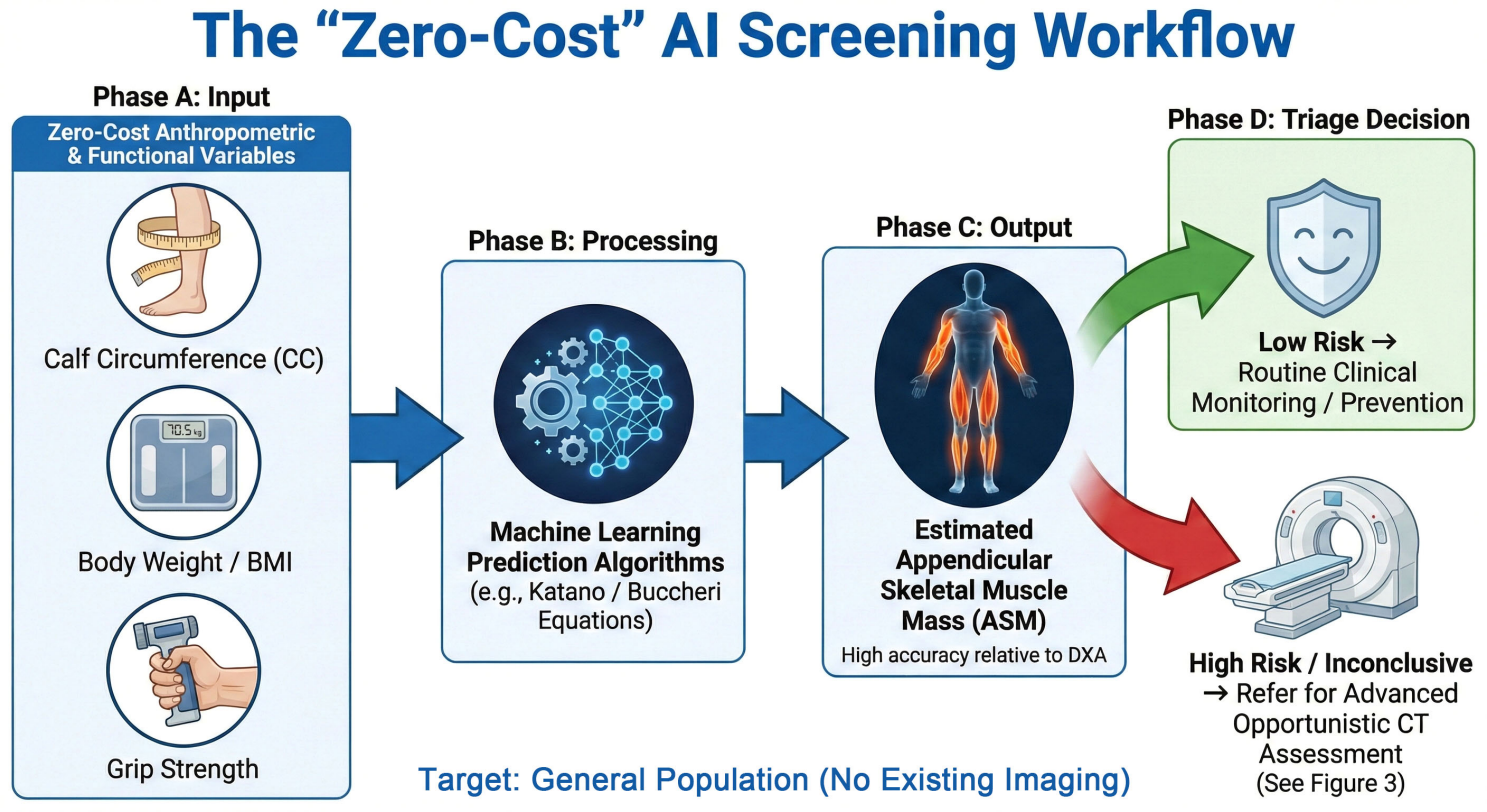
Workflow of the “zero-cost” AI screening strategy (target: individuals without existing imaging). This schematic illustrates the first-line screening approach detailed in Section 1.4. **(A)** Input: Readily available anthropometrics (Calf Circumference, Weight/BMI) and grip strength serve as zero-cost input variables. **(B)** Processing: Validated ML algorithms (e.g., Katano et al. and Buccheri et al. equations) integrate these inputs to predict muscle metrics. **(C)** Output: The model estimates Appendicular Skeletal Muscle Mass (ASM) with high accuracy relative to DXA. **(D)** Triage: Based on the estimate, “Low Risk” patients undergo routine monitoring, while “High Risk” patients are referred for advanced opportunistic CT assessment (see **Figure 3**), optimizing resource allocation.

While the EWGSOP2 guidelines emphasize muscle strength, the objective quantification of muscle mass—a requisite for confirming sarcopenia—remains resource-intensive (2). Traditional methods like DXA are often inaccessible in routine primary care (14). Artificial intelligence (AI) offers a transformative solution to these diagnostic hurdles. Specifically, machine learning (ML), a subset of AI focused on predictive modeling, addresses this barrier by utilizing simple, ‘zero-cost’ anthropometric variables to estimate muscle metrics with high accuracy (19). Recent studies highlight the value of these simplified approaches as effective initial screening tools. Katano et al. developed novel prediction equations for appendicular skeletal muscle mass using basic clinical variables, demonstrating their utility in daily practice (20). Similarly, Buccheri et al. validated that ML algorithms incorporating readily available data, such as body weight and somatic circumferences, can accurately estimate DXA-derived values through user-friendly equations (19, 21). Furthermore, González-Martin et al. confirmed that ML models based on anthropometric measurements provide a robust, non-invasive alternative for predicting low muscle mass index (22). These accessible ML tools complement advanced imaging by acting as a first-line filter, particularly for individuals without existing cross-sectional imaging data, enabling scalable screening before patients undergo the comprehensive CT or MRI assessment discussed in subsequent sections (**Figure 2**).

### 1.5 From manual to opportunistic: the AI shift in body composition analysis

Manual skeletal muscle segmentation on Computed Tomography (CT), which requires experts to meticulously delineate muscle boundaries slice-by-slice, is labor-intensive (taking 15–20 minutes per patient) and impractical for routine care (23). Deep learning–based convolutional neural networks (CNNs), which use multi-layered filters to automatically recognize image patterns such as muscle boundaries and textures, now enable automated segmentation with Dice scores >0.94 (24). By reducing processing time from hours to less than one second, these approaches enable automated, opportunistic screening based on routinely acquired CT data, without the need for dedicated or additional imaging examinations (25). Fully automated pipelines show excellent agreement with manual analysis, with <4.5% deviation in muscle area (24). Quality-control mechanisms maintain robustness across populations and protocols (24). Automated methods also quantify visceral, subcutaneous, and intermuscular adipose tissue, while transfer learning improves data efficiency (26, 27). Collectively, these advances support a shift from manual analysis toward scalable, data-driven opportunistic assessment of body composition for sarcopenia evaluation in geriatric care (23).

Therefore, the specific aim of this narrative review is to summarize the technical progress of AI-driven sarcopenia assessment and evaluate its clinical utility in geriatric orthopedics. Relevant literature was identified through searches of PubMed and Web of Science databases using keywords including ‘sarcopenia’, ‘artificial intelligence’, ‘deep learning’, and ‘geriatric orthopedics’.

## 2 Technical framework of AI-based muscle analysis

### 2.1 Automated muscle segmentation

The third lumbar vertebra (L3) vertebral level is the accepted gold-standard landmark for estimating whole-body skeletal muscle mass from single-slice CT (28, 29). Cross-sectional muscle area at L3 shows the strongest correlation with total muscle volume (r = 0.832–0.986), explaining 69–97% of variance (30, 31). This region represents peak spinal muscle area and reliably reflects body composition across sex and body size (30, 32). The L3 skeletal muscle index (area/height²) is widely validated for sarcopenia diagnosis, with cutoffs of <55 cm²/m² for men and <39 cm²/m² for women (29, 33).

Convolutional neural networks, particularly U-Net architectures—which utilize a U-shaped encoder-decoder structure with skip connections to preserve high-resolution details—dominate automated skeletal muscle segmentation (25). These models achieve Dice similarity coefficients—a metric ranging from 0 (no overlap) to 1 (perfect match) that quantifies segmentation accuracy—of >0.95 at L3, comparable to expert annotation (24, 25). Advanced frameworks such as DeepLabV3+ and UNETR further improve individual muscle delineation, with UNETR reaching Dice scores of 0.84 for thigh muscle (34). Key muscle groups including the psoas, erector spinae, and paravertebral muscles can be segmented with high reproducibility (25). Fully convolutional pipelines enable end-to-end analysis in under one second, supporting opportunistic sarcopenia screening (24).

### 2.2 Assessing muscle quantity vs. quality

Muscle quantity is traditionally evaluated through cross-sectional area (CSA) measured at the third lumbar vertebra (L3) level, with skeletal muscle index (SMI) calculated by normalizing CSA to height (35). Deep learning models achieve excellent automated segmentation of skeletal muscle with pooled Dice similarity coefficients exceeding 0.94, enabling rapid and reproducible CSA quantification (23). Alternative vertebral levels including L1 and thoracic regions demonstrate strong correlations with L3 measurements when the standard level is unavailable (35, 36).

Muscle quality, particularly in the context of myosteatosis—the pathological accumulation of adipose tissue within skeletal muscle—is evaluated using AI-based CT attenuation analysis (37). This method quantifies muscle radiodensity in Hounsfield Units (HU), where lower attenuation values indicate higher intramuscular fat content. Normal attenuation muscle area (NAMA; +30–150 HU) declines with age, while low-attenuation muscle and intramuscular fat progressively increase (37, 38). The NAMA/total abdominal muscle area index provides superior diagnostic accuracy, with sex-specific thresholds of 66.4% (men) and 65.1% (women) (37). Convolutional neural networks enable fully automated assessment with Dice values >0.97, supporting comprehensive sarcopenia evaluation (39).

### 2.3 Multi-modal data processing: handling variability between CT, MRI, and ultrasound imaging

**Figure 3:**
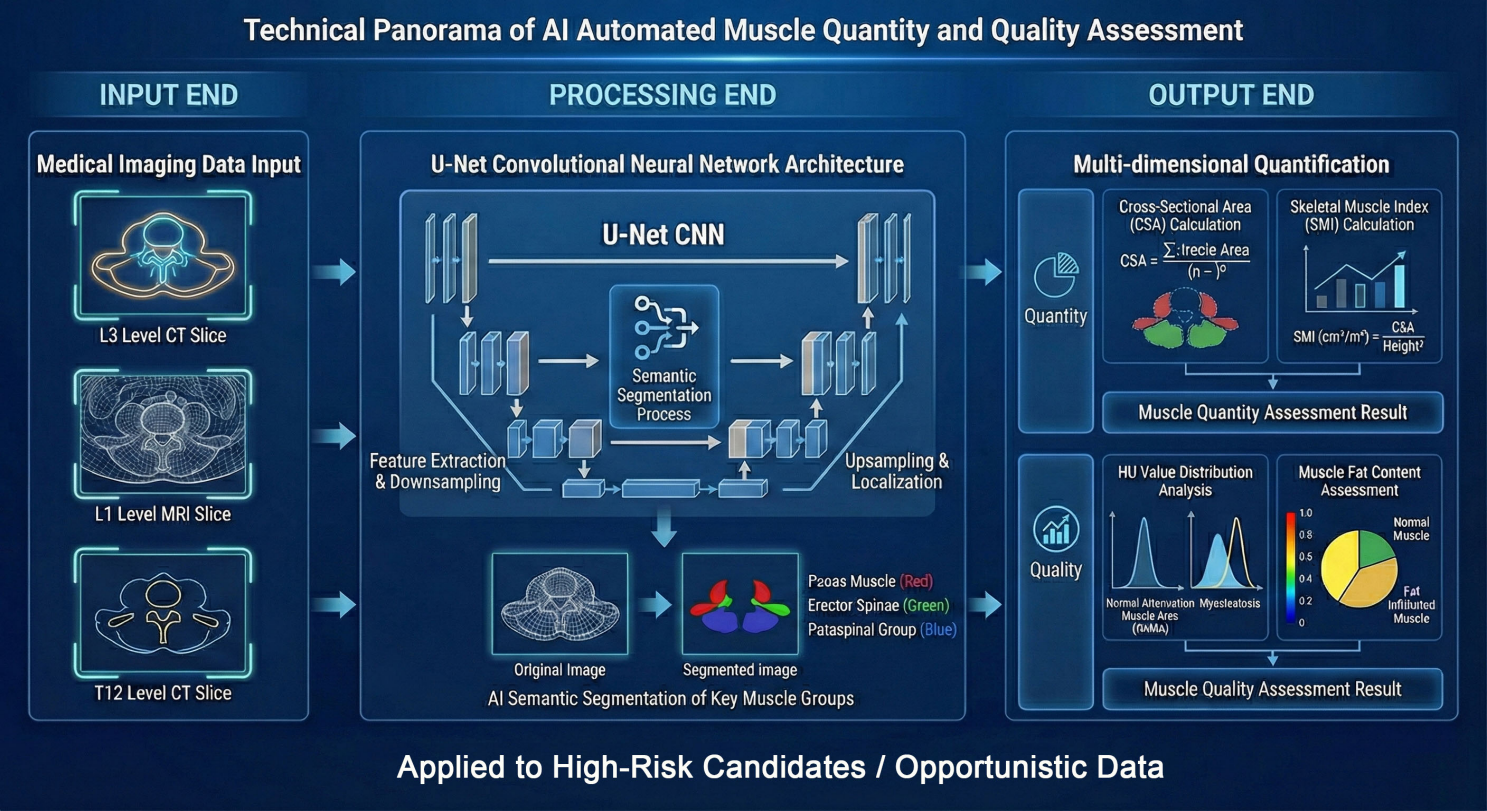
Technical panorama of the AI-based automated muscle assessment framework. The workflow illustrates a deep learning pipeline divided into three main stages: Input End: Medical imaging data, consisting of CT or MRI slices at varying vertebral levels (e.g., L3, L1, T12), serve as the input for the model. Processing End: A U-Net Convolutional Neural Network (CNN) architecture performs semantic segmentation. Through pathways of feature extraction, downsampling, upsampling, and localization, the network automatically isolates key muscle groups. The example shows the segmentation of Psoas (red), Erector Spinae (green), and Paraspinal (blue) muscles. Output End: The segmented data is transformed into multi-dimensional quantitative metrics. Quantity is assessed via Cross-Sectional Area (CSA) and Skeletal Muscle Index (SMI), calculated as CSA normalized by height squared (cm^2^/m^2^). Quality is evaluated through Hounsfield Unit (HU) value distribution analysis (distinguishing normal muscle from myosteatosis) and muscle fat content assessment. Note: While this framework achieves high-precision analysis for confirming sarcopenia, it is resource-intensive. Therefore, within the proposed stepwise diagnostic strategy, this comprehensive assessment is primarily indicated for opportunistic use (leveraging existing scans) or for “High-Risk” patients identified during first-line screening (as detailed in **Figure 2**), ensuring efficient healthcare resource allocation.

Multi-modal integration remains a major challenge in AI-based sarcopenia assessment because CT, Magnetic Resonance Imaging (MRI), and ultrasound differ technically across platforms and protocols (40). CT and MRI show strong interchangeability for skeletal muscle quantification, with correlation coefficients >0.93 for cross-sectional area and r=0.997 for T2-weighted MRI versus CT, which provides superior soft-tissue contrast, with only 0.74% bias (41, 42). However, standardized thresholds for myosteatosis have not been unified across modalities, limiting routine use (40). Deep-learning models address modality variation through transfer learning and domain adaptation (34). Ultrasound muscle thickness correlates moderately with CT-derived muscle mass (r=0.56) (43). Machine-learning fusion improves diagnostic accuracy, with score-level fusion reaching 83.17% accuracy (44). Linear regression enables CT-to-MRI fat-fraction conversion, while unsupervised clustering such as Gaussian Mixture Models performs best on CT (AUC = 0.990) compared with MRI (AUC<0.85) (45). The overall workflow of this AI-based automated muscle assessment framework is illustrated in **Figure 3**.

## 3 Opportunistic screening in orthopedic scenarios

### 3.1 Trauma & hip fractures: utilizing emergency pelvic/abdominal CT scans to predict post-operative recovery and mortality

Opportunistic CT screening utilizes routine emergency imaging to assess sarcopenia without extra radiation or cost (46, 47). Pelvic and abdominal CT performed for trauma evaluation provide prognostic skeletal muscle metrics (48). Psoas attenuation on preoperative CT shows strong mortality prediction in hip fracture patients (AUC 0.839; sensitivity 84.2%) (49). Skeletal muscle index at T12 independently predicts one-year mortality in elderly hip fracture cohorts (50). CT-defined sarcopenia increases inpatient (RR 1.96), 30-day (RR 1.60), and one-year mortality risk (RR 3.11) after trauma (51). Muscle quality markers, particularly gluteus medius and maximus attenuation, correlate with postoperative ambulation and functional recovery (52). Reduced psoas area predicts mortality and identifies sarcopenia in hip fracture patients (53). Deep-learning automation enables rapid muscle assessment at L1 or L3, broadening screening during routine imaging (33).

### 3.2 Degenerative spine surgery: using MRI/CT to assess paravertebral muscle atrophy and its link to “failed back surgery syndrome”

Paravertebral muscle degeneration is an important imaging biomarker for predicting poor outcomes after degenerative spine surgery (54). Preoperative multifidus fatty infiltration, characterized by the accumulation of intramuscular adipose tissue, is associated with higher postoperative disability scores and persistent low back pain (standardized mean difference 0.33, 95% CI 0.16–0.50) (54). Quantitative MRI demonstrates that ≥50% multifidus fatty infiltration reduces surgical success (37.5% vs. 53.7%) over five-year follow-up (55). Novel T2-weighted MRI indices, including the paraspinal muscle quality score, show that elevated lean muscle signal intensity independently predicts pain severity (odds ratio 4.88–6.07) (56). CT-based assessments further indicate that erector spinae fatty infiltration above 31.9% significantly decreases postoperative pain improvement (odds ratio 0.91) (57).

Automated deep learning algorithms enable accurate paravertebral muscle segmentation with Dice coefficients up to 0.903, supporting opportunistic screening (58, 59). Paraspinal sarcopenia correlates with long-term sagittal imbalance and functional disability after multilevel fusion, with multifidus atrophy, defined as a reduction in muscle cross-sectional area or volume, emerging as an independent risk factor for suboptimal outcomes (60).

### 3.3 Elective arthroplasty (hip/knee): pre-operative sarcopenia screening as a tool for surgical risk stratification and personalized rehabilitation

Preoperative sarcopenia is a critical risk factor in elective hip and knee arthroplasty, with prevalence ranging from 10.6% to 48.7% depending on diagnostic criteria and assessment methods (4, 61). Severe sarcopenia significantly increases the risk of delayed functional recovery following total hip arthroplasty (adjusted OR 2.82, 95% CI 1.03–7.72) (61). In total knee arthroplasty, sarcopenic patients exhibit 1.8-fold higher complication rates and 3.66-fold greater transfusion requirements than non-sarcopenic controls (4). Opportunistic CT-based muscle assessment at vertebral levels such as L3, L4, or T12 allows reliable preoperative screening without additional radiation (46, 62). Radiographic thigh muscle measurements correlate strongly with psoas muscle area and predict sarcopenia with high specificity (89–100%) (62).

**Figure 4:**
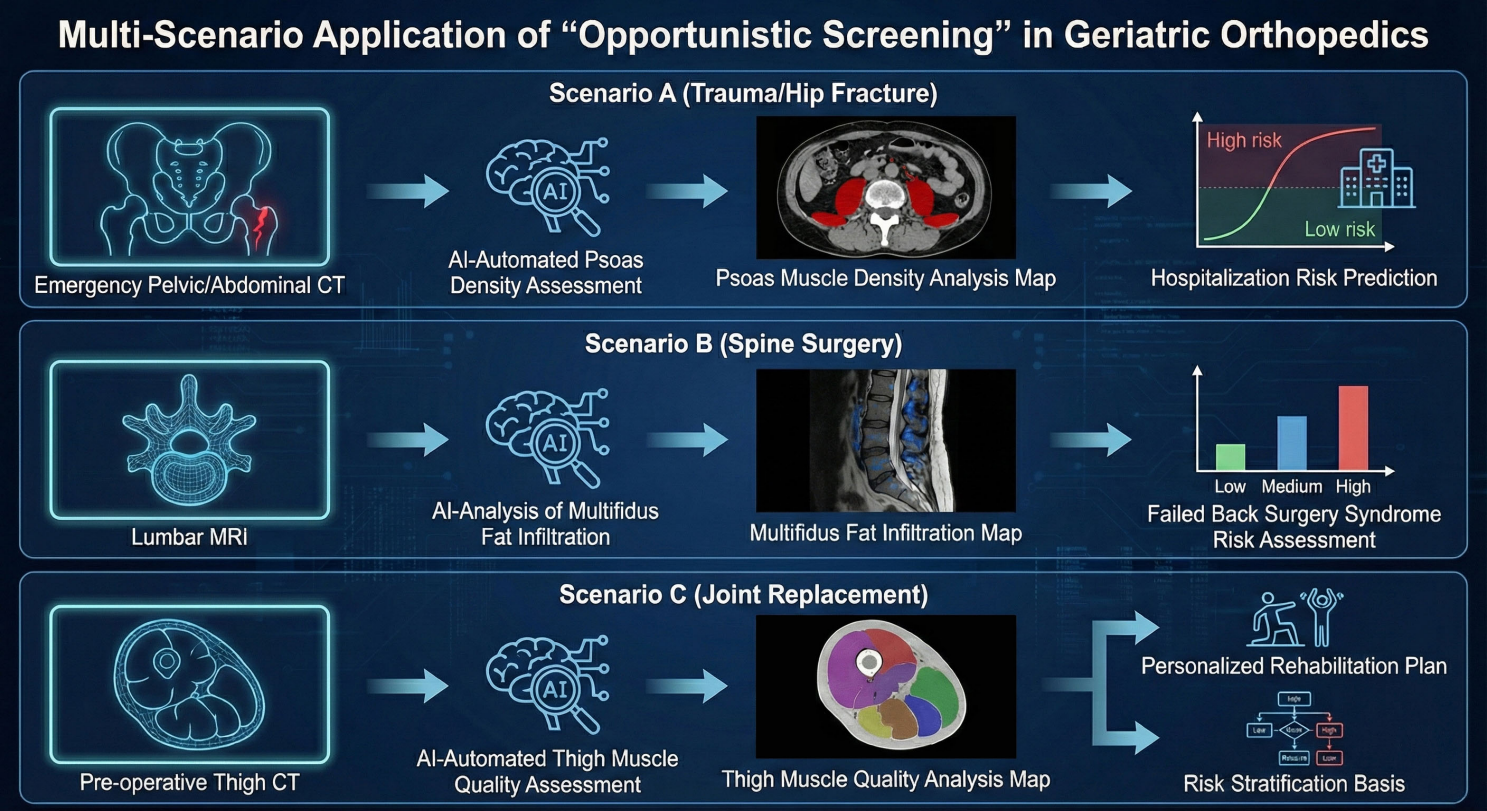
Multi-scenario applications of AI-based opportunistic screening in geriatric orthopedics. This schematic illustrates the clinical utility of automated muscle analysis across three major orthopedic subspecialties using existing diagnostic imaging. Scenario A (Trauma & Hip Fracture): In the emergency setting, AI automatically assesses psoas muscle density from routine pelvic or abdominal CT scans. This quantitative data serves as a biomarker for frailty to predict risks of prolonged hospitalization and post-operative complications. Scenario B (Spine Surgery): AI-driven MRI analysis quantifies fatty infiltration (myosteatosis) within the multifidus muscles. This assessment is used to evaluate the risk of Failed Back Surgery Syndrome (FBSS) and guide surgical decision-making. Scenario C (Joint Replacement): Pre-operative thigh CT scans are utilized by AI to evaluate muscle quality and mass. These metrics provide a basis for personalized rehabilitation protocols and perioperative risk stratification for patients undergoing total hip or knee arthroplasty. These “opportunistic” workflows leverage existing imaging data to provide high-precision, automated risk assessment without requiring additional radiation exposure or clinical costs.

Beyond perioperative complications, sarcopenia adversely influences rehabilitation, with affected patients showing inferior knee function scores at six months and slower gait speeds after arthroplasty (4, 63). Simple screening tools such as the SARC-F (Strength, Assistance with walking, Rise from a chair, Climb stairs, and Falls) questionnaire can identify at-risk individuals even before muscle mass deficits are confirmed (63). Early recognition enables targeted prehabilitation strategies incorporating resistance exercise and nutritional support, potentially improving surgical resilience and accelerating recovery (64). The multi-scenario clinical applications of AI-based opportunistic sarcopenia screening across orthopedic subspecialties are summarized in **Table 1** and further illustrated in **Figure 4**.

**Table 1:** Summary of AI-driven sarcopenia assessment across orthopedic subspecialties.

Clinical scenario	Imaging modality	AI architecture	Key muscle metrics	Performance (dice score)	Clinical implications and predictive value	References
Trauma & Hip Fracture	Pelvic/Abdominal CT	U-Net	SMI, Psoas Attenuation	> 0.94	Predicts 30-day (RR 1.60) and 1-year mortality (RR 3.11)	(5, 24)
Degenerative Spine	Lumbar MRI/CT	CNN/UNETR	Multifidus Fatty Infiltration	Up to 0.903	Associated with higher disability scores and persistent low back pain	(54, 58)
Joint Replacement	Pre-op Thigh CT	Deep Learning	CSA, Intermuscular Adipose Tissue	0.94 - 0.97	Predicts delayed functional recovery (OR 2.82)	(39, 61)
Geriatric ER	T12/L1 Level CT	Fully Automated Pipeline	SMI, NAMA (Quality Index)	> 0.94 ^13^	Independent predictor of prolonged LOS and infection	(50, 65)
Point-of-Care	Portable Ultrasound	AI-enhanced Software	Muscle Thickness/Quality	High Accuracy	Ideal for repeated bedside monitoring in immobilized patients	(43, 66)

AI, artificial intelligence; CNN, convolutional neural network; CSA, cross-sectional area; CT, computed tomography; ER, emergency room; LOS, length of stay; MRI, magnetic resonance imaging; NAMA, normal attenuation muscle area; OR, odds ratio; Pre-op, preoperative; RR, relative risk; SMI, skeletal muscle index; U-Net, U-shaped network.

## 4 Clinical predictive value of AI metrics

### 4.1 Surgical outcomes: correlation between AI-detected sarcopenia and increased length of stay, surgical site infections, and readmission rates

AI-driven sarcopenia assessment provides a robust predictor of adverse surgical outcomes in geriatric orthopedic patients. Automated CT-based body composition analysis using deep learning has emerged as an objective frailty biomarker associated with multiple postoperative complications (67). AI-identified sarcopenia is linked to prolonged hospitalization, increasing length of stay from 15 to 26 days in emergency abdominal surgery and extending stay after total knee arthroplasty and lumbar spine procedures (68, 69). AI-measured sarcopenia also predicts infectious complications, with lower muscle indices associated with higher prosthetic joint infection risk and increased 30- and 90-day septicemia and pneumonia incidence (70, 71).

Hospital readmission risk is similarly elevated. Malnutrition-sarcopenia syndrome confers a 7.64-fold increase in 6-month readmission, while lumbar arthrodesis patients demonstrate a 24% higher one-year readmission rate (72, 73). Fully automated deep learning tools measuring muscle attenuation at the L1 vertebral level show predictive performance comparable to established clinical risk scores, supporting incorporation of AI-based sarcopenia assessment into routine perioperative risk stratification (33, 67).

### 4.2 Functional recovery: predicting long-term gait speed and independence post-surgery

Preoperative sarcopenia significantly predicts diminished postoperative gait speed and functional independence in geriatric orthopedic patients (6, 74). Sarcopenic patients demonstrate slower walking speeds and increased fall risk following total knee arthroplasty, with functional deficits persisting beyond six months postoperatively (4). Machine learning algorithms utilizing preoperative sensor-derived gait metrics achieve excellent discrimination (AUC 0.80–0.86) in predicting functional recovery trajectories after knee replacement (75). Deep learning models analyzing inertial measurement unit data predict postoperative mobility and discharge destination with 82% accuracy in older surgical patients (76).

CT-derived muscle composition parameters, particularly thigh cross-sectional area and intermuscular adipose tissue, correlate strongly with gait speed recovery at six months post-hip fracture (77). Severe sarcopenia independently predicts delayed functional recovery (adjusted OR 2.82) and poorer patient-reported outcomes following total hip arthroplasty (61). Machine learning models incorporating baseline muscle mass, handgrip strength, and cognitive status demonstrate superior predictive performance (AUC 0.74–0.89) compared to traditional regression approaches for rehabilitation outcomes (78). Appendicular skeletal muscle mass loss exceeding 4.6% correlates with significant declines in activities of daily living one year after hip fracture surgery (79).

Overall, these findings highlight the pivotal role of sarcopenia and its quantitative assessment in predicting postoperative functional recovery in orthopedic patients.

### 4.3 Mortality prediction: AI muscle markers as independent predictors of 1-year mortality in geriatric trauma

AI-derived muscle metrics provide strong prognostic value for 1-year mortality in geriatric trauma, with both muscle quantity and quality acting as independent predictors (80). CT-based skeletal muscle index at T12 independently predicts 1-year postoperative mortality in elderly hip fracture patients (OR 0.881, 95% CI 0.784–0.991) (50). Automated deep-learning sarcopenia assessment at L1 shows comparable performance to L3, with 5-year mortality hazard ratios of 3.25 and 3.58 (33). Muscle density offers superior predictive capacity, with adjusted hazard ratios of 1.83–1.98 for 1-year mortality in hip fracture cohorts (81). Sarcopenia identified through opportunistic CT screening confers a 9.4-fold increased 1-year mortality risk in older trauma patients (48).

The psoas muscle cross-sectional area adjusted for height and weight (CAAST measurement) represents the strongest predictor of 6-month postdischarge mortality (HR 4.77, 95% CI 2.71-8.40) in elderly fall victims (82). Combined assessment of muscle size and density provides additive prognostic information, with both parameters independently associated with mortality risk across multiple anatomical measurement sites (83).

Altogether, assessment of both muscle quantity and quality—especially at key vertebral levels or using CAAST measurements—provides strong predictive value for short- and medium-term mortality in geriatric trauma and orthopedic patients. Combining multiple muscle metrics further improves prognostic accuracy and supports AI-driven risk stratification and personalized perioperative management.

### 4.4 From predictive analytics to actionable insights: AI-enabled clinical decision support (CDSS)

Integration of AI-derived musculoskeletal metrics into clinical workflows represents a paradigm shift from reactive fracture management to proactive population-level prevention (84). Automated body composition algorithms can be seamlessly embedded within Picture Archiving and Communication Systems (PACS) to generate “zero-click” structured reports that require no additional radiologist effort (65). These AI-enabled systems automatically quantify skeletal muscle mass, bone mineral density, and visceral adiposity from routine abdominal CT scans, triggering real-time alerts within electronic medical record (EMR) platforms when sarcopenia thresholds are exceeded (85, 86). Clinical decision support systems enhanced by AI can reduce workflow friction while generating structured data to drive downstream multidisciplinary care pathways (85).

**Figure 5:**
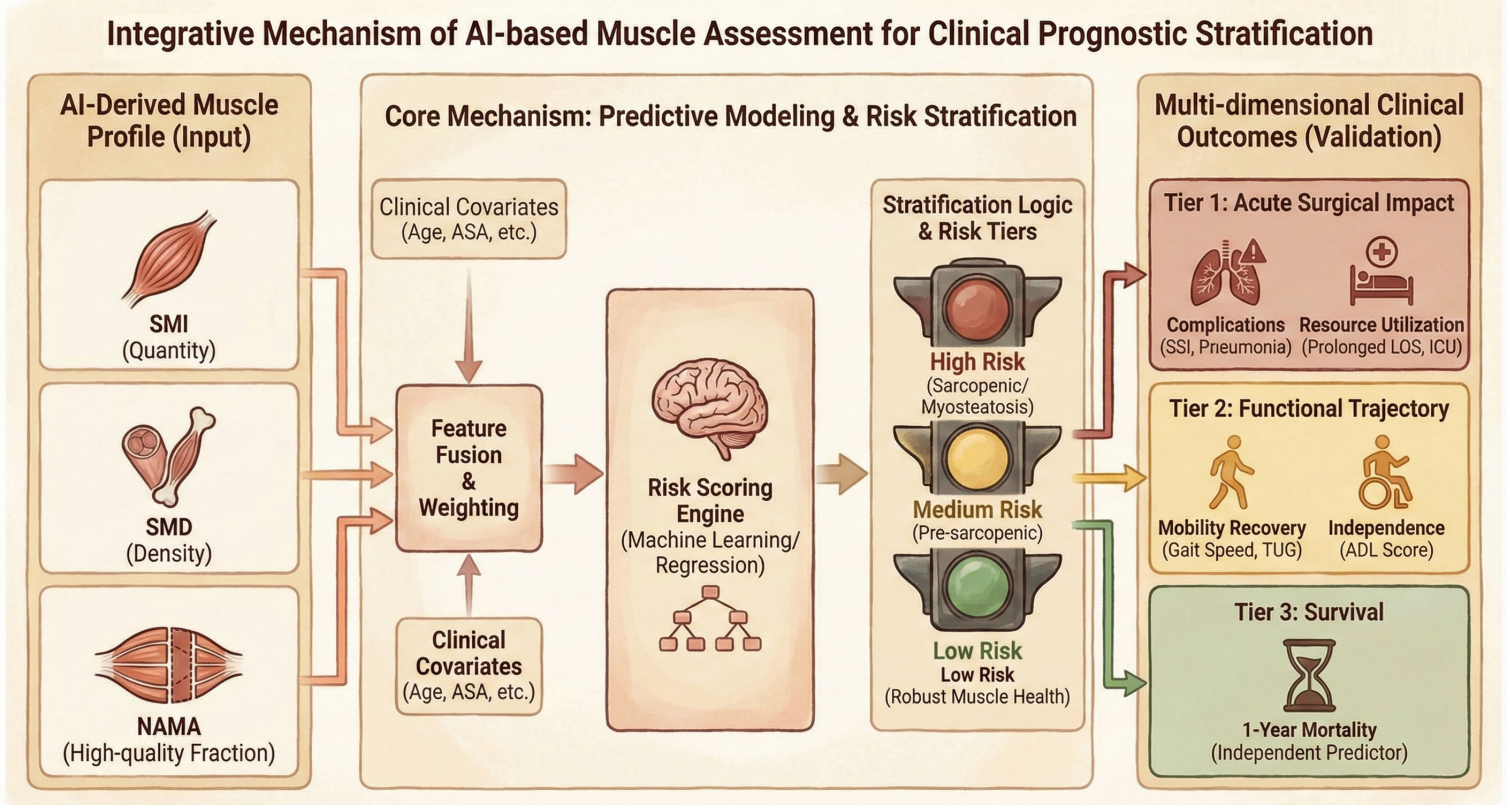
Integrative mechanism of AI-based muscle assessment for clinical prognostic stratification. AI-Derived Muscle Profile (Input): Automated extraction of muscle quantity (SMI) and quality metrics (SMD, NAMA) serves as the foundational data. Core Mechanism: Predictive Modeling & Risk Stratification: Muscle biomarkers are integrated with clinical covariates through feature fusion. A risk scoring engine then stratifies patients into High (Red), Medium (Yellow), and Low (Green) risk tiers. Multi-dimensional Clinical Outcomes (Validation): The risk stratification predicts outcomes across three tiered endpoints: Tier 1 (Acute Surgical Impact), Tier 2 (Functional Trajectory), and Tier 3 (Survival), enabling personalized perioperative management.

Automated EMR alerts improve preventive care adherence, with studies showing 2.6-fold higher screening rates (87). AI-assisted opportunistic CT screening for sarcopenia is cost-saving and clinically superior, enabling systematic identification of at-risk patients during routine imaging (86). Integrated CDSS reporting notifies primary care providers, specialists, and care navigators, supporting timely nutritional counseling, exercise programs, and fall prevention (88–90). Implementation challenges include updating guidelines, ensuring EHR interoperability, and balancing automation with clinical judgment (91, 92). Emerging evidence indicates that population-level AI screening can shift geriatric orthopedic care from episodic fracture treatment to continuous risk stratification and prevention (**Figure 5**).

## 5 Current challenges and limitations

### 5.1 Technical bottlenecks: lack of standardized hu thresholds for “low muscle quality” across different CT vendors

Substantial interscanner variability in Hounsfield unit (HU) measurements remains a major barrier to standardized muscle quality assessment (93, 94). Lamba et al. showed that HU measurements for identical soft tissues differed significantly between two major CT manufacturers across all anatomical regions (94). Sande et al. reported interscanner HU variability of 7–56 HU depending on model and energy setting, with shifts up to 79 HU when modifying tube voltage on the same scanner (93). These variations are especially problematic for muscle density assessment, as CT technical parameters such as tube voltage, contrast phase, reconstruction algorithm, and scanner type markedly influence attenuation metrics (95).

Lower kilovoltage settings increase measured muscle density by 14–40%, while intravenous contrast raises density by 6–23% in the arterial phase and 19–57% in the venous phase (96). Phantom studies indicate that although muscle area based on −29 to 150 HU thresholds is stable across protocols, muscle quality indices using mean density or narrow HU windows are inconsistent across CT settings (97). The coefficient of variation for uncalibrated HU values across scan positions reaches 6.52%, versus 0.33% with internal calibration (98). As a result, fixed HU cutoffs for low muscle quality may cause major diagnostic misclassification, particularly for myosteatosis, where inter-enhancement agreement κ values range from only 0.331 to 0.635 (99).

### 5.2 Biological variability: differences in muscle distribution across ethnicities, genders, and age groups

Biological variability presents a major challenge for artificial intelligence-driven sarcopenia assessment in orthopedic geriatrics, as muscle distribution and composition differ significantly across ethnicities, genders, and age groups (100, 101). Studies have shown that Asian populations often exhibit lower muscle mass and different cut-off values for sarcopenia compared to non-Asian cohorts, necessitating region-specific diagnostic criteria such as those from the Asian Working Group for Sarcopenia (100, 101). Gender-based differences in muscle quality and distribution further complicate AI model generalizability, with women typically having lower absolute muscle mass and distinct fat infiltration patterns (102, 103). Age-related changes, including accelerated muscle loss and altered muscle architecture, introduce additional heterogeneity that AI algorithms must account for to avoid misclassification (104, 105).

Machine learning models may inadvertently propagate bias if training datasets lack adequate representation of diverse demographic groups (103, 106). Moreover, socioeconomic factors and comorbidities, which vary by population, can confound muscle assessment and risk prediction (102, 106). The lack of universally accepted diagnostic thresholds across populations impedes the development of robust, transferable AI tools (100, 107). Standardization efforts by international working groups, such as EWGSOP and the Asian Working Group for Sarcopenia (AWGS), aim to address these disparities but remain limited by biological variability (100, 108). Ultimately, integrating multimodal data and population-specific calibration is essential for accurate, equitable AI-driven sarcopenia assessment (103, 106).

In summary, effective AI-driven sarcopenia assessment requires careful consideration of biological diversity, demographic representation, and standardized, population-specific calibration to ensure accurate and equitable evaluation across orthopedic geriatric populations.

### 5.3 Implementation hurdles: integration into the clinical workflow and the “black box” nature of deep learning models

Workflow integration represents a critical barrier to clinical adoption of AI-driven sarcopenia assessment tools (109). Successful implementation requires seamless incorporation into existing radiology information systems and electronic health records, yet most institutions lack the technological infrastructure to manage multiple AI algorithms simultaneously (110). The automated segmentation process, while achieving excellent technical performance with Dice similarity coefficients exceeding 0.94, demands standardized data exchange protocols to enable interoperable communication between AI platforms and clinical systems (23). Time constraints in busy geriatric orthopedic practices further complicate deployment, as clinicians require rapid, actionable results rather than complex computational outputs (111).

The “black box” nature of deep learning models poses substantial challenges for clinical acceptance and regulatory compliance (112). Unlike traditional statistical methods, convolutional neural networks operate through millions of interconnected parameters whose decision-making processes remain opaque to clinicians (113). This lack of interpretability undermines physician trust and complicates informed consent, as patients cannot fully understand how their imaging data influences diagnostic conclusions (112, 114). The absence of explainability also hinders error attribution when AI-assisted diagnoses prove incorrect, raising medicolegal concerns about accountability (115). Emerging explainable AI techniques, including uncertainty quantification and attention mapping, may partially address these limitations, though they often sacrifice predictive accuracy for transparency (116).

Overall, successful clinical adoption of AI-driven sarcopenia assessment requires not only seamless workflow integration but also transparent, interpretable models. Addressing technical, operational, and explainability challenges is essential to build physician trust, ensure patient safety, and facilitate regulatory compliance. Emerging explainable AI approaches offer promising solutions, yet balancing predictive accuracy with transparency remains critical for translating AI tools into routine geriatric orthopedic care.

### 5.4 Clinical applicability and resource optimization: a stepwise approach

While AI-driven analysis of abdominal CT offers high precision, it is crucial to acknowledge that such imaging is not routinely required for all orthopedic presentations, particularly for isolated upper extremity injuries or elective procedures where radiation exposure is unwarranted. To optimize healthcare resources, we propose a stepwise diagnostic workflow.

First-line screening should utilize cost-effective, non-radiating tools. As discussed in Section 1.4, machine learning algorithms based on anthropometric variables (e.g., calf circumference, BMI) combined with grip strength assessment can effectively filter the general population to identify individuals ‘truly at risk’ of sarcopenia (19–22).

Advanced AI imaging should then be reserved for specific indications: 1. Opportunistic Screening: For patients undergoing CT for primary trauma evaluation (e.g., hip fractures, polytrauma), AI algorithms can automatically extract muscle metrics from existing scans without additional cost or radiation (41, 42). 2. Diagnostic Necessity: In high-risk patients, such as those with acute hip fractures, where pain and mobility restrictions render DXA positioning impossible, CT-based AI assessment becomes a clinically justified gold standard for confirming sarcopenia and guiding perioperative management (14, 15).

This tiered approach ensures that deep learning technology is applied ethically—maximizing diagnostic yield for high-risk groups while avoiding unnecessary imaging in low-risk populations.

### 5.5 Ethical and legal considerations: incidental findings and professional liability

AI-driven sarcopenia assessment raises ethical challenges from incidental findings during automated imaging (117). Autonomous algorithms may detect unexpected pathologies, creating clinician notification and disclosure obligations that current frameworks inadequately address (118). Ambiguous accountability, the “black box” nature of AI, and potential malpractice liability complicate consent and error management (119, 120). Most FDA-cleared AI devices lack clinical trial evidence, limiting guidance for incidental findings (119). Establishing standardized protocols, clarifying professional responsibilities, and implementing risk-based liability frameworks are essential for ethical use in geriatric orthopedic care.

**Figure 6:**
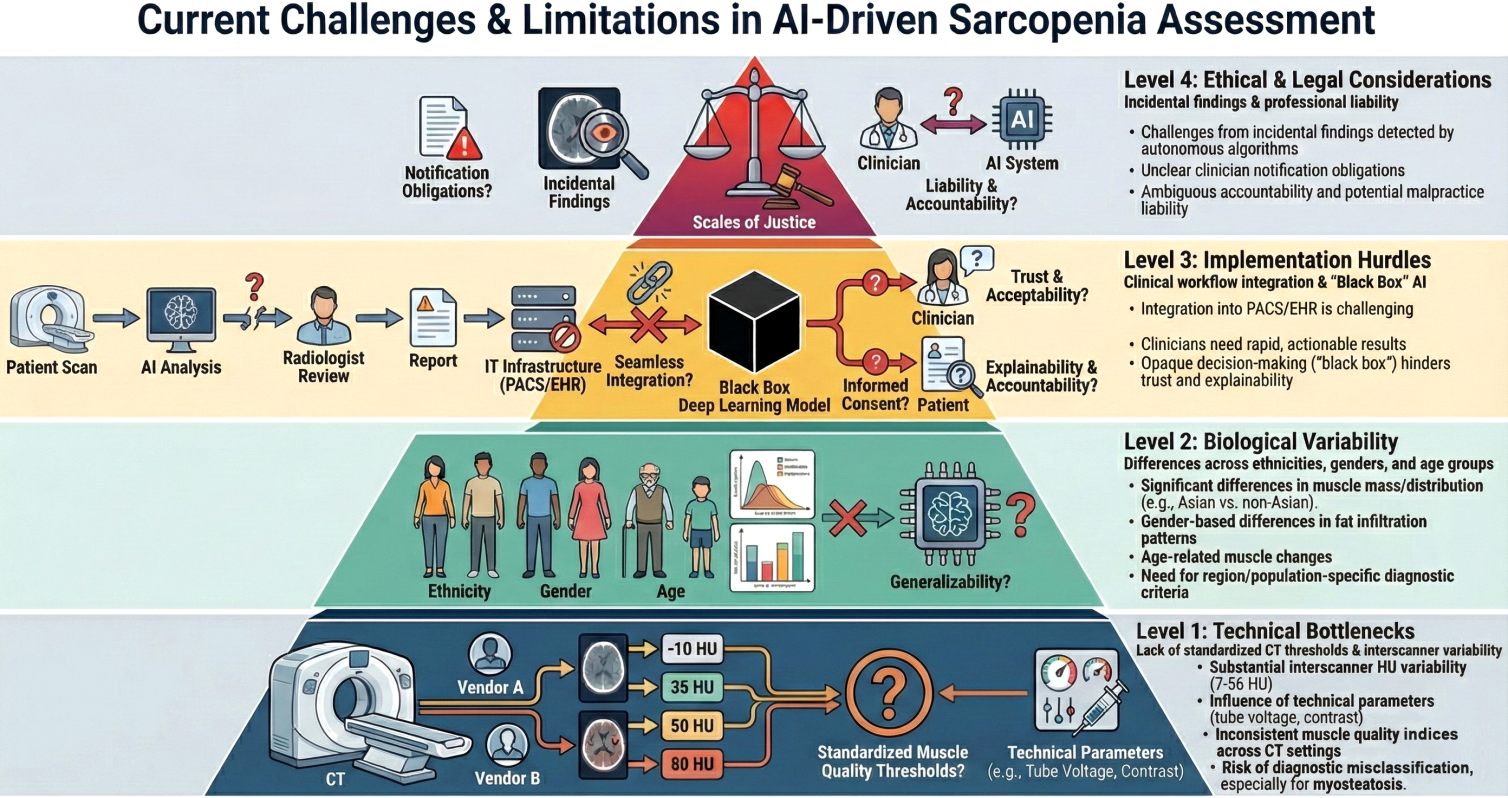
Current challenges and limitations in AI-driven sarcopenia assessment for geriatric orthopedics. Technical Bottlenecks: Large inter-scanner variability in HU values (7–56 HU, with shifts up to 79 HU) and differences in tube voltage, contrast timing, and reconstruction algorithms hinder unified diagnostic thresholds across vendors. Biological Variability: Muscle quantity, distribution, and fat infiltration differ by ethnicity, sex, and age, meaning population-specific calibration is required to avoid bias and misclassification. Implementation Hurdles: Integration of AI tools into routine PACS/EMR workflows remains difficult, and the “black-box” nature of deep learning limits interpretability, clinical trust, and informed consent. Ethical and Legal Concerns: Opportunistic screening introduces challenges regarding incidental findings, liability, and malpractice responsibility when AI contributes to diagnostic errors.

Overall, these issues underscore the substantial barriers to routine clinical adoption of AI-driven sarcopenia assessment. **Figure 6** summarizes the main challenges, including HU variability across scanners, population-dependent biological differences, workflow and interpretability limitations, and the ethical–legal consequences of opportunistic AI screening.

## 6 Future perspectives and research directions

### 6.1 Multi-modal AI fusion: combining imaging markers with clinical frailty scores and biochemical markers for a holistic “sarcopenia index”

Multi-modal artificial intelligence fusion represents a transformative approach to sarcopenia assessment by integrating heterogeneous data sources into comprehensive diagnostic frameworks (106, 121). Machine learning algorithms can synthesize CT-derived radiomics, clinical frailty indices, and biochemical markers to generate composite sarcopenia indices with superior diagnostic accuracy compared to single-modality assessments (67). Beyond current evidence, future directions include developing standardized composite indices that integrate demographic, functional, and biochemical data, leveraging advanced deep learning to extract subtle imaging features such as muscle radiodensity and intermuscular adipose tissue (67). These AI-driven holistic indices have the potential to enable precise risk stratification, guide personalized rehabilitation strategies, and support targeted interventions in geriatric orthopedic populations.

### 6.2 Longitudinal monitoring: using AI to track muscle recovery or loss during the post-operative rehabilitation phase

AI-driven longitudinal monitoring enables objective quantification of muscle changes throughout postoperative rehabilitation in geriatric orthopedic patients (122). Deep learning algorithms can efficiently track individual thigh muscle volume changes following hip fracture surgery, revealing differential recovery patterns between muscle groups (123). Machine learning models may outperform traditional approaches in predicting rehabilitation trajectories, allowing early identification of patients at risk for delayed functional recovery. AI-powered ultrasound analysis provides real-time assessment of muscle quality, while three-dimensional body composition analysis can anticipate postoperative complications (124). Serial AI-based imaging facilitates personalized rehabilitation by guiding exercise and nutritional interventions (125). To ensure reliable and scalable clinical integration, widespread implementation will require the development of standardized imaging protocols and validation across diverse geriatric populations.

### 6.3 Point-of-care AI: the potential of AI-enhanced portable ultrasound for bedside muscle assessment in orthopedic wards

Point-of-care ultrasound (POCUS) integrated with artificial intelligence represents a transformative approach for bedside sarcopenia screening in orthopedic geriatric patients (66). Technological advances in hand-held ultrasound devices enable physicians to perform rapid muscle assessments as part of routine clinical examinations (66). AI-powered software could automate muscle analysis, providing near-instantaneous, reproducible measurements (126). Deep learning models may detect sarcopenia with high accuracy, while fusion with clinical data could further enhance predictive performance (127). The portability, safety, and convenience of AI-enhanced POCUS make it an ideal tool for repeated bedside monitoring, supporting continuous rehabilitation tracking and enabling timely interventions in immobilized orthopedic patients.

**Figure 7:**
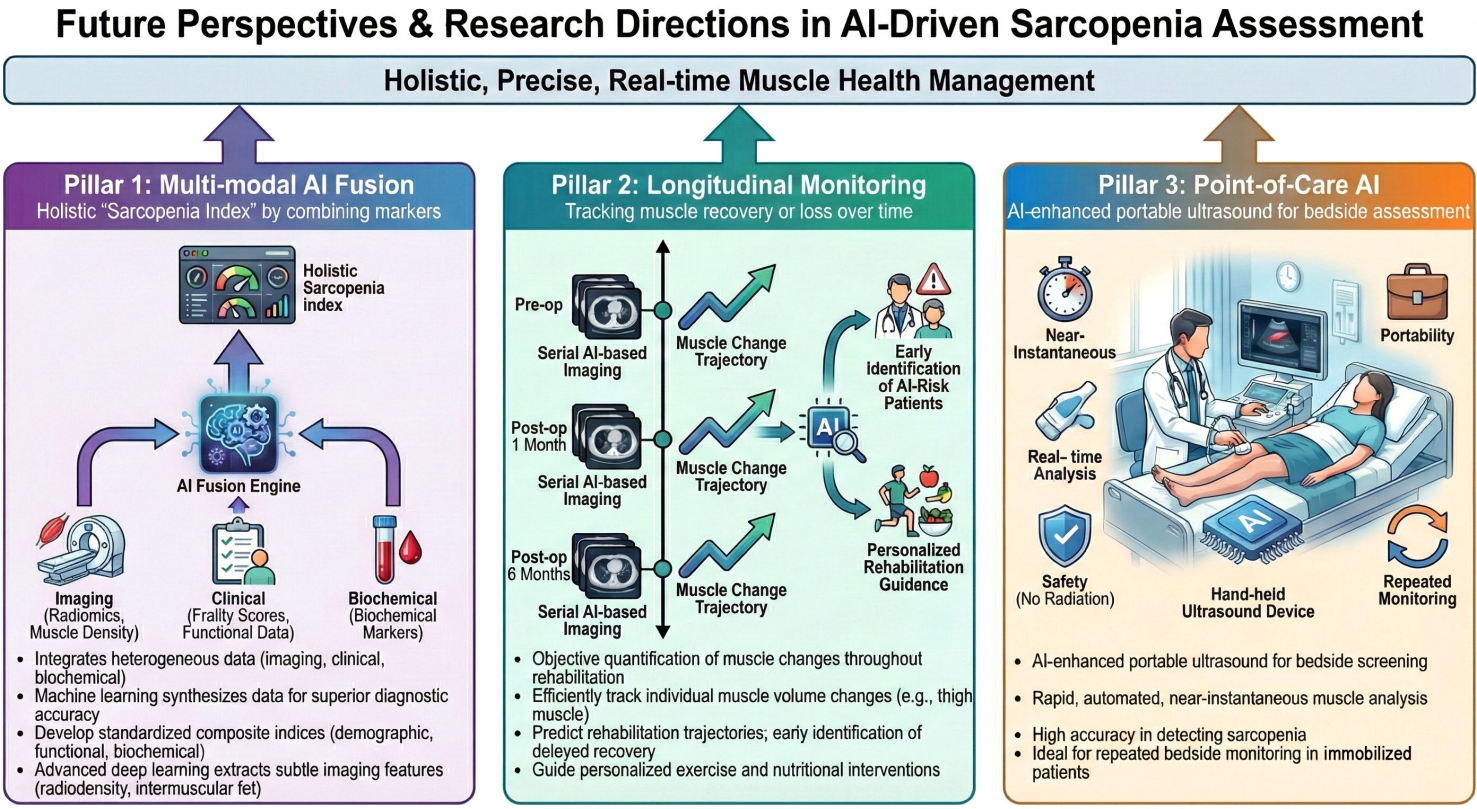
Future directions in AI-driven sarcopenia assessment. Multi-modal Fusion: Integration of imaging, clinical, and biochemical data for a holistic “Sarcopenia Index.” Longitudinal Monitoring: Tracking post-operative muscle changes and predicting rehabilitation trajectories via serial AI analysis. Point-of-Care AI: Rapid, bedside muscle screening and real-time monitoring using AI-enhanced portable ultrasound. Core Goal: Transitioning from reactive treatment to proactive, personalized musculoskeletal health management.

These advances are leading to more proactive and integrated sarcopenia care in geriatric orthopedics. As shown in **Figure 7**, key trends include multi-modal AI fusion, AI-based postoperative monitoring, and bedside ultrasound screening, supporting a shift from reactive diagnosis to personalized, preventive care.

## 7 Conclusion and clinical implications

### 7.1 The quantitative shift (diagnostic implications)

This review demonstrates that AI-driven assessment implies a fundamental shift from subjective observation to a tiered, objective diagnostic strategy. Effective management begins with “zero-cost” machine learning tools that utilize simple anthropometric variables for broad, accessible screening. Comprehensive deep learning analysis of opportunistic CT or MRI is then strategically reserved for confirming diagnoses in high-risk patients or maximizing the value of existing trauma imaging. This hierarchical approach ensures high diagnostic precision while optimizing healthcare resources and minimizing unnecessary radiation.

### 7.2 Bridging surgery and geriatric care (therapeutic implications)

A key operational implication is the integration of AI into CDSS and EMR platforms. This allows real-time alerts to multidisciplinary teams, supporting early nutritional counseling and personalized exercise interventions. Clinically, this implies a transition from reactive treatments to proactive “prehabilitation,” improving surgical resilience, accelerating recovery, and connecting acute fracture management with holistic geriatric care.

### 7.3 Long-term monitoring and challenges (prognostic implications)

AI applications, including point-of-care ultrasound, support longitudinal tracking of muscle status during rehabilitation. Despite challenges in standardization, biological variability, and interpretability, the ultimate implication of this review is that multi-modal AI frameworks offer the potential for equitable, personalized, and proactive orthopedic care for older adults.
